# Generation and Characterization of a CRISPR/Cas9-Mediated *SNAP29* Knockout in Human Fibroblasts

**DOI:** 10.3390/ijms22105293

**Published:** 2021-05-18

**Authors:** Marie Christine Martens, Janin Edelkamp, Christina Seebode, Mirijam Schäfer, Susanne Stählke, Saskia Krohn, Ole Jung, Hugo Murua Escobar, Steffen Emmert, Lars Boeckmann

**Affiliations:** 1 Clinic and Policlinic for Dermatology and Venerology, University Medical Center Rostock, 18057 Rostock, Germany; christine.martens2@med.uni-rostock.de (M.C.M.); j.edelkamp@monasteriumlab.com (J.E.); christinaseebode@web.de (C.S.); mirijam.schaefer@med.uni-rostock.de (M.S.); ole.jung@med.uni-rostock.de (O.J.); steffen.emmert@med.uni-rostock.de (S.E.); 2 Department of Cell Biology, University Medical Center Rostock, 18057 Rostock, Germany; susanne.staehlke@med.uni-rostock.de; 3 Clinic for Hematology, Oncology and Palliative Care, University Medical Center Rostock, 18057 Rostock, Germany; saskia.krohn@med.uni-rostock.de (S.K.); hugo.murua.escobar@med.uni-rostock.de (H.M.E.)

**Keywords:** CEDNIK syndrome, SNAP29, ichthyosis, CRISPR/Cas9, lentiviral transduction

## Abstract

Loss-of-function mutations in the synaptosomal-associated protein 29 (SNAP29) lead to the rare autosomal recessive neurocutaneous cerebral dysgenesis, neuropathy, ichthyosis, and keratoderma (CEDNIK) syndrome. SNAP29 is a soluble N-ethylmaleimide-sensitive factor attachment protein receptor (SNARE) protein. So far, it has been shown to be involved in membrane fusion, epidermal differentiation, formation of primary cilia, and autophagy. Recently, we reported the successful generation of two mouse models for the human CEDNIK syndrome. The aim of this investigation was the generation of a CRISPR/Cas9-mediated *SNAP29* knockout (KO) in an immortalized human cell line to further investigate the role of SNAP29 in cellular homeostasis and signaling in humans independently of animal models. Comparison of different methods of delivery for CRISPR/Cas9 plasmids into the cell revealed that lentiviral transduction is more efficient than transfection methods. Here, we reported to the best of our knowledge the first successful generation of a CRISPR/Cas9-mediated *SNAP29* KO in immortalized human MRC5Vi fibroblasts (c.169_196delinsTTCGT) via lentiviral transduction.

## 1. Introduction

Human cerebral dysgenesis, neuropathy, ichthyosis, and keratoderma (CEDNIK) syndrome (OMIM: 609528) is a rare neurocutaneous disorder that was first described in 2005 by the group of Eli Sprecher. Loss-of-function mutations in the soluble N-ethylmaleimide-sensitive factor (NSF) attachment protein 29 (*SNAP29*) gene cause this autosomal recessive disorder [1]. SNAP29 is a soluble NSF attachment protein receptor (SNARE) that is involved in membrane fusion [2], endocytic recycling [3], cell motility [4], epidermal differentiation [5,6], formation of primary cilia [7], and autophagy [1,2,4,5,6,7,8]. Children with a loss-of-function mutation in *SNAP29* usually die between the ages of 5 and 12 [9].

Several genome editing techniques exist to introduce site-specific alterations. While Zinc finger nucleases (ZFN) and transcription activator-like effector nucleases (TALEN) rely on proteins to recognize specific DNA sequences through DNA-binding domains, the clustered regularly interspaced short palindromic repeats (CRISPR)/CRISPR-associated (Cas) nuclease 9 (CRISPR/Cas9) system uses a small RNA to target a genomic location [10]. The CRISPR/Cas9 system was discovered in microbial adaptive immune response to viruses and plasmids [11]. Its use as an innovative gene editing tool was published in 2012 [12]. The method relies on a CRISPR RNA (crRNA), a trans-activating RNA (tracrRNA) and the endonuclease Cas9 to induce a site-specific double-stranded break (DSB) in the target DNA. The crRNA contains a 20-nt guide sequence complementary to the targeted DNA called protospacer. Directly downstream of the targeted DNA sequence, a protospacer adjacent motif (PAM) is required, which is a 5′-NGG using the *Streptococcus pyogenes* type II CRISPR/Cas9 system. The crRNA guides the endonuclease Cas9 to the targeted DNA in the presence of a tracrRNA. For easier use in research, a single guide RNA (sgRNA) was created fusing the crRNA and tracrRNA. The induced DSB can either be repaired by non-homologous end joining (NHEJ) or homology-directed repair (HDR). While HDR leads to accurate DNA repair, NHEJ is an error-prone DNA repair pathway that can lead to insertion-deletion (indel) mutations which may result in frameshift. This frameshift can lead to a premature stop codon [13]. This mechanism can be exploited to target specific genes of interest in order to generate knockout (KO) cell lines.

Various methods to deliver the CRISPR/Cas9 system into a cell are available. For transient expression of the sgRNA and the endonuclease Cas9, plasmid transfection using different transfection reagents can be used [13]. For permanent expression of the sgRNA and the endonuclease Cas9, lentiviral transduction is available [14,15].

While a KO animal model can be used to explore cellular interaction within an organ and interactions between organs, interactions between components of the skin can differ between animal skin and human skin. Additionally, ethical concerns and interindividual differences between animals arise with the use of animal models [16]. Furthermore, animal models are relative expensive in comparison to cell culture. All cells within the cell culture have a defined genetic background. Therefore, cell culture provides a well-defined system to study the functional role of specific genes in human cells.

To further study the role of SNAP29 in cellular homeostasis and signaling in an animal model independent manner, we generated a CRISPR/Cas9-mediated *SNAP29* KO in a human fibroblast cell line (MRC5Vi) via lentiviral transduction. The KO was confirmed by Sanger sequencing and Western blot analysis and is characterized by impaired growth compared to the parental wildtype (WT) control.

## 2. Results

### 2.1. Efficiency of Lentiviral Transduction and Plasmid Transfection in Different Cell Lines

**Figure 1:**
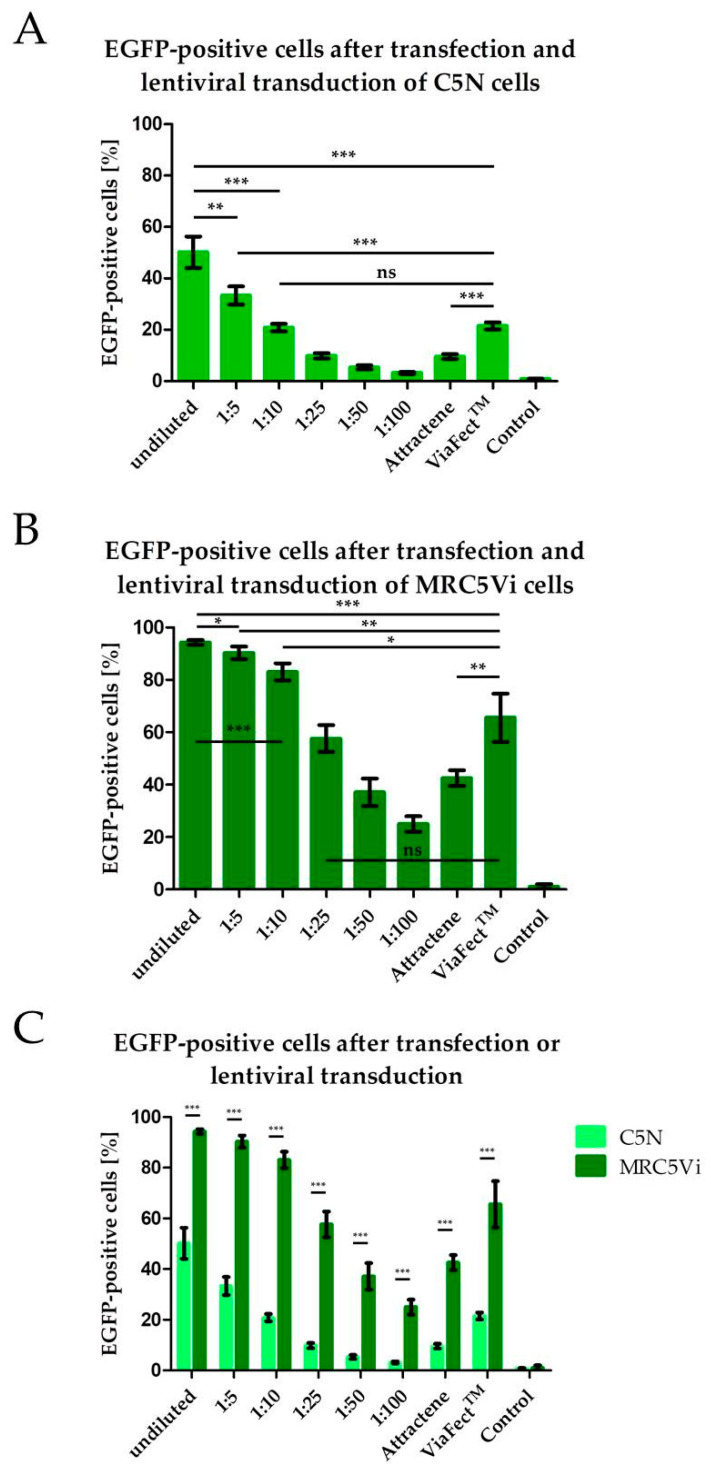
Lentiviral transduction efficiency in a human and a murine cell line compared to transfection efficiency. The cells were seeded at 100,000 cells per well in a 6-well plate and transduced 24 h later using different lentiviral vector suspension dilutions as shown in the graph. Murine C5N keratinocytes were harvested at 72 h after transduction, human MRC5Vi fibroblasts at 48 h after transduction for flow cytometry. Those would be the selected time points for puromycin selection after lentiCRISPR v2 application. To be able to compare transduction and transfection the same EGFP plasmid was used (pLJM1-EGFP). The transfected cells were harvested for flow cytometry measurements after 24 h. (**A**,**B**) show the results of C5N keratinocytes’ and MRC5Vi fibroblasts’ transduction and transfection efficiency, respectively. (**C**) compares the data of (**A**,**B**). Statistical analyses were performed using unpaired two-tailed Student’s *t*-test (*n* (transfected and transduced cells) = 4, *n* (control cells) = 7; * *p* < 0.1, ** *p* ≤ 0.01, *** *p* ≤ 0.001).

Introduction of foreign genetic information into cells can be achieved using various methods including transfection and lentiviral transduction [13,14,15]. Here, we compared various vector suspension dilutions for lentiviral transduction and plasmid transfection with two different transfection reagents, namely Attractene and ViaFect™. Additionally, we compared these methods using a human and a murine cell line by inducing EGFP expression in the cells. Using flow cytometry, cells were gated for EGFP expression. EGFP-positive cells were considered successfully transfected or transduced. Lentiviral transduction resulted in significantly more EGFP-positive cells in both murine C5N keratinocytes and human MRC5Vi fibroblasts (50.2% and 94.3%, respectively) than in transfected cells (Attractene: 9.6% and 42.6%, respectively; ViaFect™: 21.5% and 65.6%, respectively) (Figure 1A,B). Furthermore, transfection using ViaFect™ resulted in significantly more EGFP-positive cells than cells transfected using Attractene both in murine and human fibroblasts. Additionally, the amount of EGFP-positive cells decreases with decreasing concentrations of lentiviral vector concentrations used for transduction. Interestingly, fewer C5N cells were EGFP-positive compared to MRC5Vi cells after both transduction and transfection (Figure 1C, showing the combined data of Figure 1A,B). These results indicate that lentiviral transduction is more efficient than transfection and that human cells are more susceptible to gene transfer than murine cells. Moreover, the tested transfection reagents show varying efficiencies with Attractene being less efficient than ViaFect™ in the tested cell lines.

**Figure 2:**
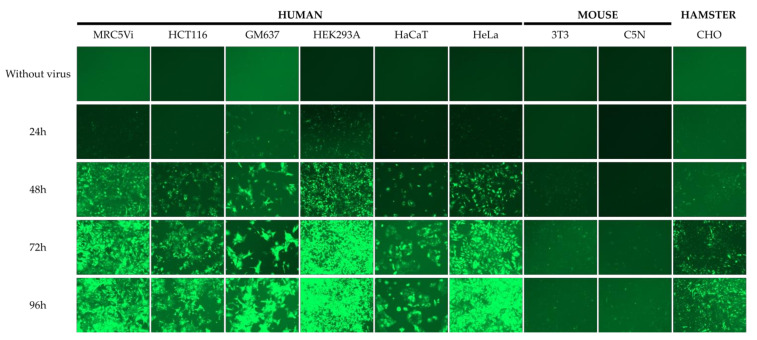
Transduction efficiency of various established cell lines using lentiviral transduction of an EGFP-coding plasmid. Viruses were produced in HEK293T cells using psPAX2, pCMV-VSVg, and pLJM1-EGFP with Attractene transfection reagent. The different cell lines were seeded at 100,000 cells/well in a 6-well plate and transduced 24 h later using the filtered virus stock. Using fluorescence microscopy, pictures were taken at the indicated time points (EGFP: 1800 ms, scale bar in the lower right: 400 µm).

To further explore the differences between the number of EGFP-positive cells following lentiviral transduction of different cell types, we transduced various established cell lines from three different species (human, mouse, and hamster). Using fluorescence microscopy, we detected more EGFP-positive cells in human cell lines than in hamster-derived and murine cell lines after transduction. Additionally, EGFP-positive cells could be detected earlier in the six used human cell lines. Interestingly, the hamster-derived cell line (CHO) showed more EGFP-positive cells than the two used murine cell lines (Figure 2). These findings suggest that lentiviral vectors show a tropism depending on the species the cells were derived from (human > hamster > mouse).

### 2.2. Generation of a Complete SNAP29 CRISPR/Cas9 KO in MRC5Vi Cells

**Figure 3:**
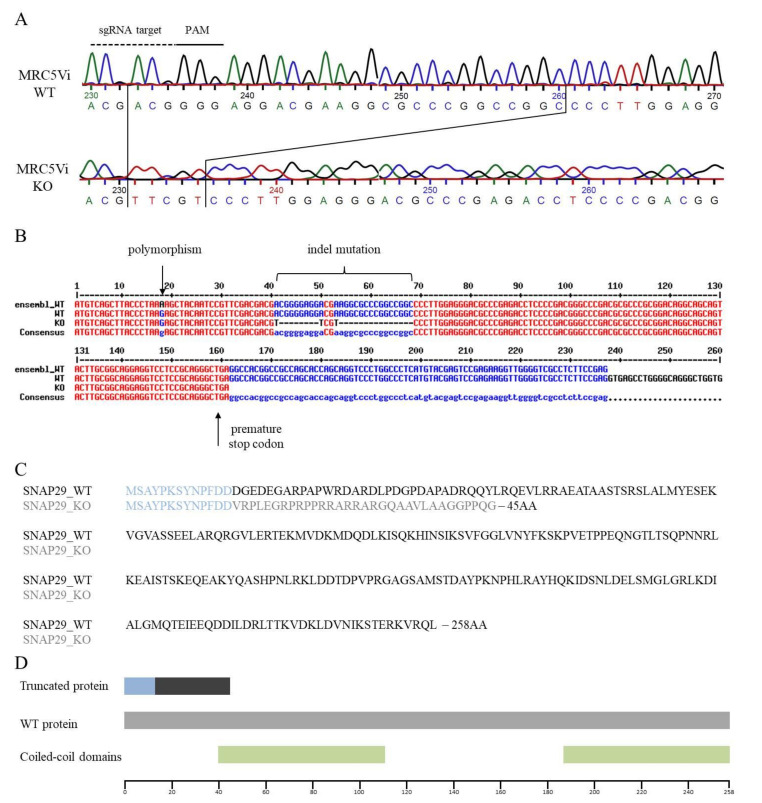
Sequencing analysis of a *SNAP29* knockout (KO) cell line compared to wildtype (WT). (**A**) The *SNAP29* KO fibroblast clone was sequenced by Sanger sequencing and compared to the WT MRC5Vi DNA sequence. The sgRNA target and the PAM sequence are highlighted. Sequence comparison showed an indel mutation, in exon 1 (c.41_68delinsTTCGT). (**B**) Further comparison showed a polymorphism on position 18 of the used MRC5Vi fibroblasts using the DNA sequence from the database Ensembl (https://www.ensembl.org, accessed on 11 November 2018) as a reference. (**C**) Comparison of the resulting protein sequence: identical sequences are shown in light blue (13 amino acids). The modified protein sequence from the KO clone is shown in light gray (32 amino acids). The resulting truncated protein contains 45 amino acids in total. (**D**) Position of functional coiled-coil domains of SNAP29: the truncated protein is presented in the colors as mentioned above.

**Figure 4:**
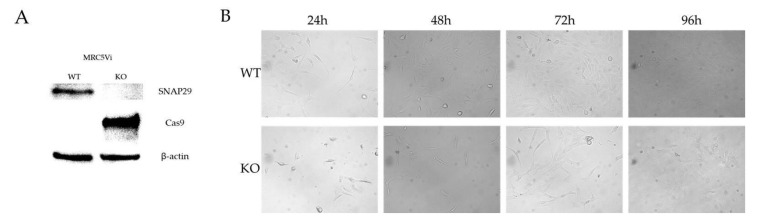
Characterization of *SNAP29* knockout (KO) fibroblasts compared to wildtype (WT) fibroblasts. (**A**) Western blot analysis was performed for MRC5Vi *SNAP29* KO and WT. Whole-cell protein was separated by polyacrylamide gel electrophoresis (PAGE) and transferred onto a nitrocellulose membrane via semi-dry Western blotting. The presence or absence of SNAP29, Cas9, and the housekeeping protein β-actin was visualized using specific antibodies (actin as control). Raw data is presented in Supplementary Material S1. (**B**) To analyze the cell growth of *SNAP29* KO cells, WT and *SNAP29* KO fibroblasts were seeded in a 6-well plate at a density of 100,000 cells per well. Cell growth was assessed every 24 h and documented by light microscopy (20×, scale bar in the lower right: 200 µm).

A homozygous knockout (KO) of *SNAP29* in the human fetal lung fibroblast cell line MRC5Vi was achieved using a lentiviral transduction-based CRISPR/Cas9 technique. Lentiviral vectors were produced in the packaging cell line HEK293T and then used for transduction of MRC5Vi cells. Successfully transduced cells were selected with puromycin and single clones separated by dilution and seeding in 96-well plates as described in material and methods. DNA from several independent clones was isolated and used for sequencing analyses. Sequencing of the targeted exon one of *SNAP29* revealed a homozygous indel mutation in one of the analyzed clones with a deletion of 28 bp and an insertion of 5 bp (c.41_68delinsTTCGT). This mutation led to a reading frame shift and a premature stop codon, terminating the coding sequence 91 nucleotides after the indel mutation. The resulting protein consisted of 45 amino acids with only the first 13 amino acids being unchanged from the wildtype (WT) protein. The functional coiled-coil domains of SNAP29 were not included in the truncated protein (Figure 3). By Western blot analyses no SNAP29 protein was detected in MRC5Vi *SNAP29* KO cells (expected band size approximately 5 kDa) compared to parental WT cells (expected band size: 29 kDa), confirming the KO of SNAP29 on the protein level. No protein was detected in the protein samples from the KO cells using the SNAP29 antibody at the aforementioned band size (Figure 4A, Supplementary Material S1). Due to lentiviral vector DNA integration into the host genome, Cas9 expression could be detected in the KO cells but not in parental cells. Although the MRC5Vi *SNAP29* KO cells are viable, growth was substantially reduced compared to the parental WT cells (Figure 4B).

## 3. Discussion

Here, we reported the successful generation of a CRISPR/Cas9-mediated *SNAP29* knockout (KO) in human fibroblasts. Sanger sequencing showed a homozygous indel mutation in exon 1 which led to a premature stop codon and, therefore, a truncated protein without the functional domains of SNAP29. The homozygous mutation can be explained by a two-stage mechanism. Since it is unlikely that the homozygous indel mutation was generated by two simultaneous deletion and insertion events in both alleles, we hypothesized that this may either be due to a loss of one allele or due to a sequential process of an initial deletion and insertion in one alleles cause by non-homologous end joining (NHEJ) followed by repair of the other alleles through homology-directed repair (HDR) using the altered allele sequence as a template.

We used lentiviral transduction to generate the KO fibroblasts. Lentiviral vectors are replication-deficient hybrid viral particles based on lentiviruses. Therefore, they are able to integrate their viral genome anywhere within the target cell genome, even within genes. Thus, they are able to influence cell functions [17,18]. Although targeted alterations may take place, other genes of the target cell genome might be affected [19]. Due to the sgRNA and the endonuclease Cas9 permanently being expressed within the target cells, off-target effects are more likely. Then again, on-target effects are also more likely leading to every transduced cell being a potential KO cell the longer the sgRNA and endonuclease are expressed. To minimize off-target effects, inducible lentiviral vectors should be preferred [20], although the problem of insertional mutation would still exist. Other non-integrating viral vectors such as adenoviral vectors could be considered alternatively [21].

Lentiviral transduction is based on receptor-mediated endocytosis or fusion of the viral membrane with the cell membrane and therefore the release of the nucleocapsid containing the viral genetic information [17]. Using pseudotyping with VSV-G, a wider spectrum of cells can be targeted due to VSV using the LDL receptor and other LDLR family members to enter a cell [22]. Still, transduction efficiency varies between species. It has been reported that some murine and rat cell lines can be transduced less efficiently [23]. Herein, we showed a lower lentiviral transduction efficiency in murine cell lines compared to human cell lines by transducing various cell lines to express EGFP. It has been discussed that this might be due to low expression of the CMV promoter in rodent cells as EGFP is under the control of a CMV promoter. Exchanging it for a EF1α promoter was shown to lead to equally high transduction efficiency in rodent cells as in human cells [23]. Other studies showed a lower expression of the human CMV promoter in murine cells compared to human cells as well [24]. Another study discusses the internalization of VSV-G pseudotyped lentiviral vectors as limitation [25]. Repeating this experiment using other promoters for EGFP such as EF1α would be beneficial to discern whether the CMV promoter or VSV-G pseudotyping can be attributed to the different transduction efficiencies. Additionally, we showed that transfection efficiency differs between transfection reagents and depends on the cells used. ViaFect™ showed a higher transfection efficiency than Attractene in both cell lines used. Since other groups had similar results showing transfection efficiency differences between different cell lines and between transfection reagents [26,27] the ideal transfection reagent should be determined for each cell line to ensure the best results.

SNAP29 has been shown to be involved in various cell functions such as endocytosis [3], autophagy [8], and formation of primary cilia [7]. Recently, a study suggested berbamine as a novel autophagy inhibitor blocking the interaction between SNAP29 and VAMP8 and thereby inhibiting autophagosome-lysosome fusion. Since berbamine showed antitumor activities in some types of cancer, it was proposed that berbamine might be used either alone or to enhance the effect of chemotherapy in cancer by modulating autophagy [28,29]. The KO cell line described here can be used to further elucidate the role of SNAP29 in autophagy and the effects of berbamine independent of its inhibition of SNAP29-VAMP8 interaction.

Primary cilia play an important role in cell proliferation, cell signaling, and tissue homeostasis [30]. A signaling pathway associated with primary cilia is the Sonic hedgehog (Shh) pathway. The transmembrane protein Patched1 (Ptch1) is located on primary cilia. Upon the binding of Shh to Ptch1, Ptch1 exits the primary cilium and the transmembrane protein Smoothened (Smo) can accumulate in the primary cilium. That, in turn, leads to the activation of Gli transcription factors [31]. A mutation in Ptch1 facilitates a permanent activation of the Shh pathway and is associated with basal cell carcinoma [32]. Since approximately 85% of basal cell carcinomas display mutations in the Shh pathway and around 73% of those are attributed to a mutation in *Ptch1* [33], the role of SNAP29 in basal cell carcinoma should be further investigated. An antitumorigenic effect of *SNAP29* KO should be considered. Further research using this KO cell line should include the introduction of the aforementioned Ptch1 mutation into the cell line to observe possible antitumorigenic effects and the influence of SNAP29 on basal cell carcinoma.

The *SNAP29* KO cell line described here will be beneficial in further elucidating the role of SNAP29 in cellular homeostasis and signaling because a direct and reproducible comparison between WT cell capabilities and *SNAP29* KO cell capabilities are now possible.

## 4. Materials and Methods

### 4.1. Cell Culture

The wildtype (WT) immortalized human fetal lung fibroblasts MRC5Vi cell line was kindly supplied by Sarah Sertic (University of Milan, Department of Life Sciences, Milan, Italy). Additionally, human embryonic kidney (HEK) 293T cells (ATTC^®^, CRL-3216™, Manassas, VA, USA), HEK293A cells (Invitrogen, R705-07, Waltham, MA, USA), human HeLa cells (ATCC^®^, CCL-2™, Manassas, VA, USA), HaCaT human keratinocytes [34], human colon HCT116 cells [35], human skin fibroblasts GM00637 (Coriell Institute, Camden, NJ, USA), mouse C5N keratinocytes (from E. H. Epstein Jr.), mouse 3T3 fibroblasts [36], and Chinese hamster ovary (CHO) cells [37] were used. Cells were cultivated in Dulbecco’s modified Eagle’s medium (DMEM) high-glucose culture media (Gibco^®^, Life Technologies, Eggstein, Germany) supplemented with 10% fetal bovine serum (FBS) (Biochrom AG, Berlin, Germany) and 1% penicillin and streptomycin (PAA, Coelbe, Germany) in a humidified atmosphere at 37 °C and 5% CO_2_. For HEK293T cells, heat-inactivated (hi) FBS was used (incubated at 56 °C for 30 min). For viral vector harvest, DMEM high-glucose culture medium supplemented with 40% hiFBS and 1% penicillin and streptomycin were used (harvest medium). Cells were passaged 1:4–1:10 when they reached confluency depending on the cells used. Therefore, cells were rinsed with 10 mL of phosphate buffered saline (PBS, pH 7.4, Merck, Darmstadt, Germany) and dissociated from the culture flask (175 cm^2^) using 4 mL trypsin/EDTA (Lonza, Basel, Switzerland) and 5 min incubation at 37 °C. The reaction was stopped by the addition of 10 mL of complete culture medium. Then, cells were centrifuged at 100× *g* for 5 min and the supernatant was discarded.

### 4.2. Lentiviral CRISPR Construct Generation

The lentiCRISPRv2 plasmid contains the cDNA encoding Streptococcus pyogenes Cas9 (hSp-Cas9) and a puromycin resistance cassette (Addgene plasmid # 52961, Watertown, MA, USA). The guide sequence oligonucleotides (5′-CACCGCAATCCGTTCGACGACGACG-3′, 5′-AAACCGTCGTCGTCGAACGGATTGC-3′), including the BsmBI restriction site overhangs, targeting the first exon within *SNAP29* were annealed (95 °C, 5 min, cool down overnight) and cloned into the lentiCRISPRv2 vector. The plasmid was digested with BsmBI (NEB, Ipswich, MA, USA) in accordance with the manufacturer’s instructions. Afterwards the ~12 kb fragment was gel purified, the ~2 kb filler piece was discarded (Gel extraction and PCR Clean Up, Macherey–Nagel, Düren, Germany). The oligonucleotides were bought 5′-phosphorylated (Sigma–Aldrich, Taufkirchen, Germany) and were ligated into the vector using the T4 Ligase (Thermo Scientific, Waltham, MA, USA) in accordance with the manufacturer’s instructions. Constructs were transformed into *Escherichia coli* strain *StbI3* cells (Invitrogen, Waltham, MA, USA) and selected using ampicillin (Sigma–Aldrich, Taufkirchen, Germany; 100 µg/mL). To validate the correct sequence, we used the BigDye^®^ Terminator v3.1 Cycle Sequencing Kit (Applied Biosystems, Foster City, CA, USA) with the U6 forward (fwd) primer 5′-ACTATCATATGCTTACCGTAAC-3′ (Sigma–Aldrich, Taufkirchen, Germany). Chromatograms were generated (Eurofins Genomics, Ebersberg, Germany) and analyzed with the Chromas Lite version 2.01 software (Technelysium Pty Ltd., South Brisbane, Australia). The guide sequence was selected using in silico on- and off-target predictions (http://crispr.mit.edu/, http://crispr.cos.uni-heidelberg.de/index.html, accessed on 13 September 2017).

### 4.3. Lentiviral Vector Production

Lentiviral vectors were produced seeding 4 × 10^6^ HEK293T cells in 100-mm tissue culture dishes (Greiner Bio-One, Frickenhausen, Germany) in 10 mL of the medium as described above. After 48 h of culture, the cells were transfected in accordance with the manufacturer’s protocol. Briefly, 0.2 µg of pCMV-VSV-G (Addgene, #8454, Watertown, MA, USA), 2 µg of psPAX2 (Addgene, #12260, Watertown, MA, USA), and either 2 µg of pLJM1-EGFP (Addgene, #19319, Watertown, MA, USA) as a control or 2 µg of lentiCRISPR v2 (Addgene, #52961, Watertown, MA, USA) were filled up using 15 µL of Attractene transfection reagent (Qiagen, Hilden, Germany) to a total volume of 300 µL of DMEM without supplements. After 24 h, the culture medium was exchanged for harvest medium. After 48 h, the supernatant was collected and cleaned up using a 0.45-µm PES filter (Sarstedt, Nümbrecht, Germany). Lentiviral vector suspension was stored at −20 °C or directly used for transduction.

### 4.4. Lentiviral Transduction

MRC5Vi fibroblasts were seeded at a density of 10^5^ cells in 1.5 mL of medium with supplements per well in 6-well plates. The following day, cells were transduced. Various lentiviral vector suspension dilutions (undiluted, 1:5, 1:10, 1:25, 1:50, 1:100) containing 15 µg of Polybrene (Sigma–Aldrich, Taufkirchen, Germany) were added to the cells cultured in 1 mL of fresh medium. For antibiotic selection, MRC5Vi cells were treated with 0.25 µg/mL of puromycin (InvivoGen, San Diego, CA, USA) after 48 h of incubation. The cells were cultured until the control cells had all died.

### 4.5. Single Clone Expansion

For single clone selection, cells were separated using the serial dilution method in a 96-well plate (Greiner Bio-One, Frickenhausen, Germany) after coating with superfibronectin (Sigma–Aldrich, Taufkirchen, Germany) and cells were further cultured in FibroLife^®^ fibroblast medium (Lifeline© Cell Technology, Frederick, MD, USA) without puromycin. After 2 days, the plate was evaluated under the microscope (Axiovert A1, Zeiss, Oberkochen, Germany); wells containing single cells were marked and cells further cultured for 2 weeks to form colonies. Then, the colonies were transferred into 6-well plates (Greiner Bio-One, Frickenhausen, Germany) and further expanded for genomic DNA isolation.

### 4.6. Sequencing and Clone Evaluation

Cells were harvested as described above and genomic DNA was isolated using the QIAamp DNA Blood Mini Kit (Qiagen, Hilden, Germany) in accordance with the manufacturer’s instructions. The genomic region of *SNAP29* exon one was amplified using the PCR primers (Sigma–Aldrich, Taufkirchen, Germany) 5′-ATGGACAGTAGGCTGCGGTT-3′ and 5′-TTTCTCGTCTCCGTTTGACAG-3′. PCR products were purified using an ExoSAP (Affymetrix, Santa Clara, CA, USA) digestion and sequenced with the BigDye Terminator v3.1 Cycle Sequencing Kit (Applied Biosystems, Foster City, CA, USA) with the PCR primer 5′- ATGGACAGTAGGCTGCGGTT-3′. Chromatograms were generated (Eurofins Genomics, Ebersberg, Germany) and analyzed with the Chromas Lite version 2.01 software (Technelysium Pty Ltd., South Brisbane, Australia) and an online multiple sequence alignment tool [38].

### 4.7. Transient Transfection for Flow Cytometry

MRC5Vi cells and C5N keratinocytes were seeded in 24-well plates (Greiner Bio-One, Frickenhausen, Germany) at a density of 1.5 × 10^4^ cells and transfected using ViaFect™ Transfection Reagent (Promega, Mannheim, Germany) and the transfection protocol in accordance with the manufacturer’s instructions (0.5 µg of pLJM1-EGFP, 1.5 µL of ViaFect™, filled up to a total volume of 50 µL Opti-MEM™ I Reduced Serum Medium, no phenol red (Life Technologies, Eggenstein, Germany)). Additionally, the two cell lines were seeded in 100-mm tissue culture dishes and transfected in accordance with the manufacturer’s protocol (4 µg of pLJM1-EGFP (Addgene, #19319, Watertown, MA, USA) using 15 µL of Attractene transfection reagent (Qiagen, Hilden, Germany) filled up to a total volume of 300 µL of DMEM without supplements).

### 4.8. Flow Cytometry

Transduced cells were harvested for flow cytometry at the time when puromycin selection would usually start (MRC5Vi fibroblasts after 48 h, C5N keratinocytes after 72 h). Transfected cells were harvested 24 h after transfection. Cells were harvested using trypsin/EDTA as described above. Cells were washed in PBS twice and afterwards resuspended in 400–500 µL of PBS. Flow cytometry was performed using FACSCalibur (BD Biosciences, Heidelberg, Germany, software CellQuest Pro 4.0.1) gating for viable cells being either enhanced green fluorescent protein (EGFP) positive or negative. The results were analyzed using the FlowJo^®^ v10 software (FLOWJO, Ashland, OR, USA).

### 4.9. Preparation for Fluorescence Microscopy

The different cell lines mentioned above were seeded at 100,000 cells per well in a 6-well plate and were transduced 24 h later using the undiluted filtered virus stock as previously described. Images were taken before transduction and 24, 48, 72, and 96 h after transduction.

### 4.10. Fluorescence and Bright Field Microscopy

For fluorescence and bright field microscopy, the Axiovert A1 microscope from Zeiss (Zeiss, Oberkochen, Germany) was used. A scale bar in the images indicates the magnification.

### 4.11. Western Blot Analysis

WT or *SNAP29* KO MRC5Vi cells were harvested as described above. Cell pellets were washed twice with 10 mL of ice-cold PBS. Cell pellets were resuspended in an appropriate volume of lysis buffer (10 mL of PBS, 1 mM of phenylmethylsulfonyl fluoride (PMSF (Sigma–Aldrich, Taufkirchen, Germany)), 1 Complete ULTRA tablet Mini EDTA (Roche, Mannheim, Germany). The suspensions in the reaction tubes were frozen in liquid nitrogen and afterwards thawed three times. Afterwards, the reaction tubes were put in an ultrasonic bath with ice for 30 min. Thereupon, the mixture was pelleted at 14,000 U/min for 30 min. The supernatant was transferred into a new reaction tube. Protein concentration was examined with a ready-to-use Bradford solution (Bradford Mix Roti^®^-Quant, Roth, Karlsruhe, Germany) and photometrically quantified. A BSA calibration curve was established using a standard serial dilution (0–1500 μg/mL). Each sample was diluted 1:20 and 1:40 in the respective buffer and 150 µL of Bradford solution (Roti^®^-Quant 1:5 in Aqua Bidest) and incubated with shaking for 5 min at room temperature. The OD595 was measured with a GloMax^®^ Discover Multimode Detection System (Promega, Mannheim, Germany). For SDS–PAGE, the Mini-PROTEAN Tetra cell and precast 4–12% polyacrylamide gradient gels (Bio-Rad, Hercules, CA, USA) were used applying the semidry-blot method in a Trans- Blot^®^ Turbo™ Transfer System (Bio-Rad, Hercules, CA, USA). Equal amounts of WT and *SNAP29* knockout (KO) MRC5Vi protein extracts were loaded onto the gel together with a prestained protein marker (Marker VI, AppliChem, Chicago, IL, USA). The WesternBreeze™ Chemiluminescent Immunodetection System for rabbit or mouse (Invitrogen, Waltham, MA, USA) was used in accordance with the manufacturer’s instructions. The anti-SNAP29 rabbit monoclonal antibody clone 111 303 (Synaptic Systems, Göttingen, Germany), anti-Cas9 mouse monoclonal antibody from Streptococcus pyogenes clone 7A9-3A3 (active motif, La Hulpe, Belgium), and anti-β-actin mouse monoclonal clone AC-74 (Sigma–Aldrich, Taufkirchen, Germany) were diluted 1:300, 0.5 µg/mL, and 1:2500 in blocking solution, respectively. Incubation with the specific antibodies was performed overnight at 4 °C. Finally, incubation with the secondary antibodies (anti-mouse or anti-rabbit) was performed for 30 min. Chemiluminescence was developed and quantified using the Chemo Cam Imager 3.2 and the LabImage 1D software (Intas, Göttingen, Germany).

### 4.12. Statistical Analyses

Statistical analyses were performed using GraphPad Prism 5.03 (GraphPad Software, La Jolla, CA, USA). Statistical significance was determined using unpaired two-tailed Student’s *t*-test (* *p* < 0.1, ** *p* ≤ 0.01, *** *p* ≤ 0.001).

## Data Availability

The raw data for analyses presented in the figures of this study are available on request from the corresponding author.

## Supplementary Materials

The following are available online at https://www.mdpi.com/article/10.3390/ijms22105293/s1, Supporting information SNAP29 Western blot.
