# Supplementary material for: Generation and Characterization of a CRISPR/Cas9-Mediated SNAP29 Knockout in Human Fibroblasts
Source: Int J Mol Sci. 2021 May 18;22(10):5293. doi: 10.3390/ijms22105293 (PMC8157373; doi:10.3390/ijms22105293)
Supplement: Supplementary file 1 [file ijms-22-05293-s001.zip › ijms-1210579-SI.pdf]

β-actin

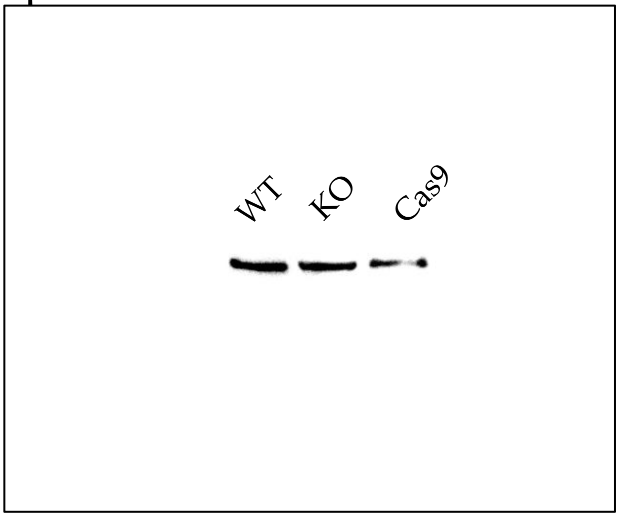

Cas9

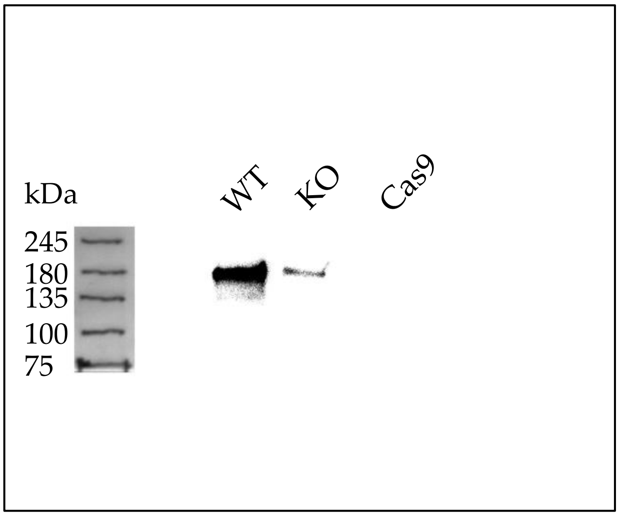

SNAP29

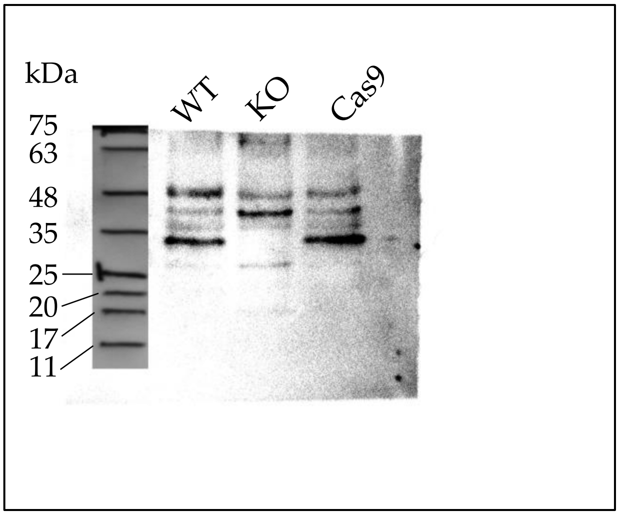

Supporting information: Western Blot Analyses

WT: MRC5Vi wildtype
KO: MRC5Vi SNAP29 knockout
Cas9: MRC5Vi Cas9 stable expression
